# Supplementary material for: Convcast: An embedded convolutional LSTM based architecture for precipitation nowcasting using satellite data
Source: PLoS One. 2020 Mar 11;15(3):e0230114. doi: 10.1371/journal.pone.0230114 (PMC7065808; doi:10.1371/journal.pone.0230114)
Supplement: Attachment — Submitted filename: Rebuttal_Letter-Final-Feb16th.pdf [file pone.0230114.s001.pdf]

Dear reviewers and academic editor,

Thank you for your review of our manuscript with the title '*Convcast: An embedded convolutional LSTM based architecture for precipitation nowcasting using satellite data* '. Your valuable suggestions and comments have helped us to improve our paper. We have incorporated almost all suggestions by the reviewers. We agree with all your suggestions but at some places our motivation was little different which we have stated here. In the following part of this letter, we reply to your original comments, which we included in red font, corresponding to the revision in the manuscript.

On behalf of all co-authors,
Yours sincerely,
Ashutosh Kumar

Comments from the Academic Editor:

Please be more experimentally convincing in answering the questions as to why and how Convcast outperforms the state-of-the-art ConvLSTM. Also, please clarify the components of the model and their interaction.

We would like to clarify that our intention is to come up with an architecture for precipitation nowcasting from *satellite data* instead of using *Ground-based radar data* which most of the previous researches have focused on; as also commented by the reviewer #2. In this sense, we do not wish to outperform the ConvLSTM. In fact, our Convcast architecture consists of several layers of ConvLSTM. ConvLSTM based architecture has been shown to be successful in precipitation nowcasting using *Ground-based radar data* which motivated us to develop an architecture from ConvLSTM cells for nowcasting using *satellite data*. However, finding a network architecture for a new problem always requires a lot of trial and errors because of several hyperparameters involved and this is where we make use of the novel Automated Machine Learning (AutoML) to obtain the Convcast architecture which outperforms several optical flow baseline methods on the test dataset as shown experimentally in the paper. Use of AutoML is a novel study area and this research is one of the very first ones that incorporates AutoML for precipitation nowcasting from satellite data.

We have now included another ConvLSTM based architecture (please see response to the reviewer #1) for more experimentally convincing the superiority of Convcast and the effect of kernel size and filters on the validation loss. We also have now better clarified the components of the model and their interaction.

We note that [Figure(s) 1,2,7] in your submission contain [map/satellite] images which may be copyrighted. All PLOS content is published under the Creative Commons Attribution License (CC BY 4.0), which means that the manuscript, images, and Supporting Information files will be freely available online, and any third party is permitted to access, download, copy, distribute, and use these materials in any way, even commercially, with proper attribution. For these reasons, we cannot publish previously copyrighted maps or satellite images created using proprietary data, such as Google software (Google Maps, Street View, and Earth). For more information, see our copyright guidelines: http://journals.plos.org/plosone/s/licenses-and-copyright.

We require you to either (1) present written permission from the copyright holder to publish these figures specifically under the CC BY 4.0 license, or (2) remove the figures from your submission.

After talking to co-authors, I came to know that no map file is used. Rather, the plots are generated by us, and coastal lines are simply overlaid. No licenses are involved here. All map files and images are produced by us and coming directly from NASA's IMERG data and they have already been projected to grids. There is no involvement of any type of license and all plots are produced by us.
The dataset used in the visualization has now been cited in the manuscript in accordance with data citation format mentioned on the NASA Precipitation Measurement Missions website https://pmm.nasa.gov/data-access/citations. The reference number is [28] in the revised manuscript as shown below:

"Huffman GJ, Bolvin DT, Nelkin EJ, et al.. Integrated Multi-satellitE Retrievals for GPM (IMERG); 2014. ftp://arthurhou.pps.eosdis.nasa.gov/gpmdata/. Available from: http://dx.doi.org/10.5067/GPM/IMERG/3B-HH/05."

Comments from the Reviewer #1

The only comparison with pure ConvLSTM approach presented in table 6 fails to isolate the reason why Convcast outperforms ConvLSTM. Are three layers better than one? Maybe the 3D convolution is crucial? Or is it because of hyperparameters, and the ConvLSTM baseline is simply crippled because of small 2x2 filters (original paper uses 3x3 and 5x5)? It is unfortunate these questions can't be answered based on the provided experiments.

We wanted to show the extreme cases and that is why we compared 2 x 2 ConvLSTM with the Convcast which has 7 x 7. In the discussion, we wrote that the accuracy of the model increases with the increase in kernel size because a larger kernel has a larger receptive field. We have now included a 5 x 5 baseline in Table 6 to show the effect of larger kernel on decreasing loss. 3D Convolution is present in all the ConvLSTM networks which has been used to get precipitations from the last ConvLSTM layer's activations before it passes through ReLU to suppress any negative values to zero. More deeper networks are better than shallow networks. However, in our research all ConvLSTM baselines have the same backbone as the Convcast. Thus, it is because of the right choice of the hyperparameters obtained from the AutoML experiment which makes the Convcast outperform other ConvLSTM baseline methods. We have made the required changes in the manuscript as shown below:

Results

Table 6. Comparison of validation loss of Convcast with other LSTM models

Models	Validation loss
LSTM (-2048-1024-[22,500])	1.569
ConvLSTM (2x2-32-2x2-32-2x2-32-1x1x1)	0.822
ConvLSTM (5x5-64-5x5-64-5x5-64-1x1x1)	0.626
**Convcast**	**0.6049**

“The loss of ConvLSTM based model decreases with the increase in the kernel size (5 x 5) and the number of filters (64). Convcast achieves the minimum loss on the validation set.”

Discussions

“The accuracy of the ConvLSTM model increases with increasing kernel size and the number of filters because a large kernel has more receptive field and can capture more spatial information and larger number of filters can have more number of feature maps. Since the ConvLSTM based architectures as shown in Table 6 have same backbone in terms of number of layers and activation functions as Convcast, the least validation loss obtained by Convcast is due to the tuned hyperparameters obtained from the AutoML experiment.”

I was confused by the description of dichotomous method (usually called binary classification in ML-related texts).

This is a common terminology in weather forecast/nowcast. We have provided the proper definitions of dichotomous method (binary classification) and would like to keep it the same way. Most of the papers on forecasting/nowcasting use the same terminology and we would like to maintain the consistency. It has now been clarified in the text as shown below:

“Accuracy indices based on dichotomous (binary classification) method”
“The first method is based on the dichotomous approach (binary classification), where we convert the predicted and observed matrix to a binary matrix consisting of zeros and ones.”

I am used to neural networks trained for classification tasks with softmax or sigmoid activation functions attached to the last layer. In this familiar setting, the output is almost always greater than zero and binarization by comparison with zero is not appropriate, some other threshold have to be chosen. As far as I understand in case of Convcast, ReLU was chosen as the activation function on the last layer, so negative activations turn into zeros (no rain prediction) and it all makes sense both in terms of resulting classification metrics and RMSE. I believe this point should be clarified in the text.

Yes, in classification related tasks with softmax or sigmoid activation functions, the output value (probability) will be greater than zero in most of the cases and binarization is made using a threshold greater than zero (generally 0.5).
As mentioned by you, the output of our last layer is not probability rather precipitation value, and thus we have chosen ReLU in the last layer. In fact, an activation function in the last layer might not be necessary (we can use y = x) as it is totally a regression problem. However, since we know from our experience that precipitation cannot take negative values, we put ReLU as an activation function at the last layer to turn negative activations to zero. Again, the output of ReLU can be any value greater than or equal to zero. We have now clarified this point in the text:

“In the last layer of the Convcast architecture, we use ReLU as the activation layer. This is because precipitation nowcasting is a regression problem where the output of the Convcast is a precipitation value. Since precipitation cannot take negative values, we choose ReLU to turn any negative activations into zeros (i.e. no rain).”

The notation at line 110 is confusing, use \times{} instead of X

Thank you for pointing it out. We have replaced X with \times{}.

~~$X \in R^{MXNXZ}$~~ $X \in R^{M \times N \times Z}$

<u>Comments from the Reviewer #2</u>

The paper proposed a new architecture for precipitation nowcasting called Convcast. Rather than using the radar echo data, Convcast utilizes the satellite data for precipitation nowcasting. Satellite data are able to reflect rainfall at a higher altitude and are available for the entire globe. Thus, studying the precipitation nowcasting problem with the help of satellite data is a valuable research topic. The Convcast model is based on Convolutional LSTM (ConvLSTM). The author proposed to use automatic machine learning (AutoML) to search for the network structure, which is a nice approach to automate the trial-and-error phase in designing deep learning models. The author illustrates that Convcast achieves state-of-the-art performance by the experiments.

The paper is well written and is a nice addition to the recent trend of incorporating machine learning to solve environmental problems.

Thank you for your comments!
